# Effect of physiotherapy rehabilitation on osteogenesis imperfecta with a midshaft tibial fracture in the 11-year-old patient: a case report

**DOI:** 10.11604/pamj.2022.43.201.34702

**Published:** 2022-12-22

**Authors:** Radha Manish Nangliya, Deepak Satish Jain, Akshaya Virendra Saklecha, Deepali Swapnil Patil

**Affiliations:** 1 Ravi Nair Physiotherapy College, Datta Meghe Institute of Medical Sciences, Sawangi (Meghe), Wardha, Maharashtra, India,; 2 Department of Musculoskeletal Physiotherapy, Ravi Nair Physiotherapy College, Datta Meghe Institute of Medical Sciences, Sawangi (Meghe), Wardha, Maharashtra, India

**Keywords:** Osteogenesis imperfecta, brittle bone disease, midshaft tibial fracture, physiotherapy rehabilitation, case report

## Abstract

Osteogenesis imperfecta (OI), a brittle bone disease is a rare genetic condition characterised by skeletal anomalies that results in higher bone fragility, reduced bone mass, deformity, and other connective-tissue signs in which the body is unable to form healthy bones. This case report presents a case of an 11-year-old male kid who visited our hospital with a complaint of pain and deformity in his left leg. After investigations, he was diagnosed with osteogenesis imperfecta with a midshaft tibial fracture of the left leg. Physical therapy rehabilitation was started and plays one of the important roles in the management of this condition along with medical and orthopedic management. Physical therapy involves strengthening exercises, stretching exercises, bracing, functional activities, gait training, etc. This case study highlighted that physical therapy rehabilitation along with multidisciplinary care; can help the patient with pain management and functional independence which enhances the patient’s strength, endurance, prevents deformity, and improves the patient’s quality of life.

## Introduction

Osteogenesis imperfecta is an uncommon genetic disease characterized by skeletal defects that cause bone fragility, bone mass loss, deformity, and other connective-tissue signs [1]. This condition is also called brittle bone disease [2]. Mutations in one of the two genes producing collagen type 1 cause of the illness in the majority of individuals, although no such mutations are discovered in some. One in 10,000 to 20,000 persons worldwide suffer from osteogenesis imperfecta, which means that this condition is rare. It affects both men and women equally, as well as all ethnic groups. Patients with osteogenesis imperfecta exhibit defects in proteins with a wide variety of functions, from structural to enzymatic, and from intracellular transport to chaperones [3]. FRAX is a fracture risk algorithm that considers femoral neck bone mineral density as determined by dual-energy X-ray absorption spectroscopy (DXA) [4]. Physical examination may reveal skeletal deformities due to unrecognised fractures (e.g., loss of height, kyphosis, or reduced rib-pelvis space), identify possible secondary causes of skeletal fragility (e.g., blue sclera with osteogenesis imperfecta, urticarial pigmentosa with systemic mastocytosis, dermatitis herpetiformis with celiac disease, or bone tenderness), and identify possible secondary [5]. Osteogenesis imperfecta is defined clinically by a wide spectrum of symptoms and severity. In addition to skeletal abnormalities, it can cause dental and craniofacial deformities, muscular weakness, hearing loss, respiratory and cardiovascular problems [6]. Secondary characteristics of OI include macrocephaly, hearing loss, basilar invagination, and cardiac issues [7]. Multiple fractures and progressive bone abnormalities, such as long bone bowing and scoliosis, can occur in infancy as a result of this. The disease was diagnosed by X-ray, CT-scan, blood examination, physical examination. The advent of bisphosphonate therapy for moderate to severe cases of osteogenesis imperfecta is the most significant therapeutic development [1]. For patients with osteogenesis imperfecta, multidisciplinary care enhances their quality of life. Physical therapy, medical treatment, and, if necessary, orthopedic surgery are all part of the treatment. Physical therapy includes pain management, strengthening, muscle stretching, gait training.

## Patient and observation

**Patient information:** an 11-year-old boy visited Acharya Vinoba Bhave Rural Hospital (AVBRH) with the complaint of pain and deformity in his left leg. He was apparently alright 15 days back then he had trauma while playing; sustaining injury to the left leg which was sudden in onset and gradually progressive pain was associated with deformity over both legs since birth. The pain was not radiating to any other body part. It does not have any diurnal or seasonal variation. It´s aggravated by movements and relieved by rest. Pain is dull aching in nature. He was unable to talk since birth. He was admitted for the same complaint at Bhandara Hospital and was referred to AVBRH for further management. At AVBRH, he was operated on for cleft palate on 7^th^ December 2021. The history of the patient was, he had a global developmental delay with macrocephaly with intellectual disability. He had a history of trauma one year ago on the same leg. There were no comorbidities. The investigation was done which revealed a midshaft tibial fracture on the left side. And he was managed by open reduction and internal fixation with plate osteosynthesis and referred to physiotherapy.

**Figure 1:**
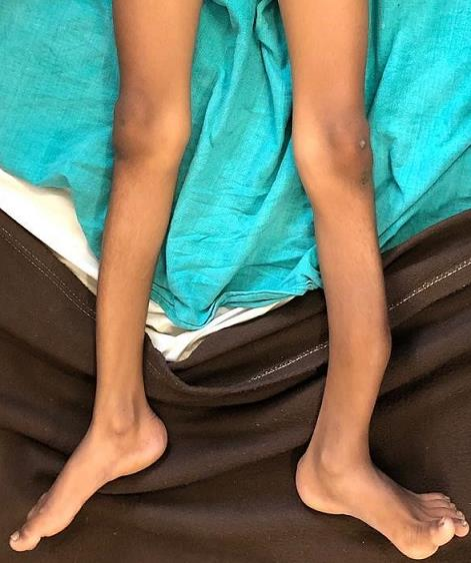
patient was in supine position showing angular curvature of tibia of left leg

**Figure 2:**
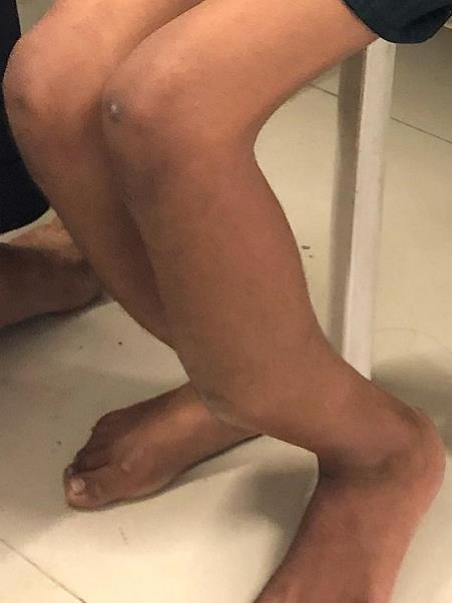
patient was in sitting position showing anterior bowing of tibia of the left leg

**Clinical findings:** the patient was evaluated in supine position (Figure 1), with a tibial fracture in his left leg. The attitude of the limb was hip and knee in neutral position with patella facing upwards. The swelling was present over the fractured limb. The left lower limb shows osteogenesis imperfecta, with shortening of the lower limb and angular curvature of the tibia and fibula, as shown on radiographic examination. The temperature, pulse rate, and BP were normal on physical examination, with a pulse rate of 90 beats per minute, blood pressure of 130/90mmHg and a respiratory rate of 24 breaths per minute. On inspection, anterior bowing of tibia was present (Figure 2) and healed scar mark present over the fracture site and overlying skin appears normal with no dilated veins, discharging sinus and mild bowing of right leg is also observed with apparent limb length discrepancy and thoracic curvature scoliosis was seen. On palpation, there was a rise in local temperature. Tenderness was present over the midshaft tibia and over the fracture site with abnormal mobility and intact sensations.

**Timeline of the current episode:** on 12^th^ December 2021, he was injured while playing. On 3^rd^ December 2021, he was admitted to AVBRH for management. On 4^th^ December 2021, he was diagnosed with osteogenesis imperfecta and midshaft tibial fracture in his left leg. On 7^th^ December 2021, he was operated for cleft palate. On 13^th^ December 2021, he was operated for the osteotomy and open reduction internal fixation with plating. On 16^th^ December 2021, physiotherapy rehabilitation was started.

**Figure 3:**
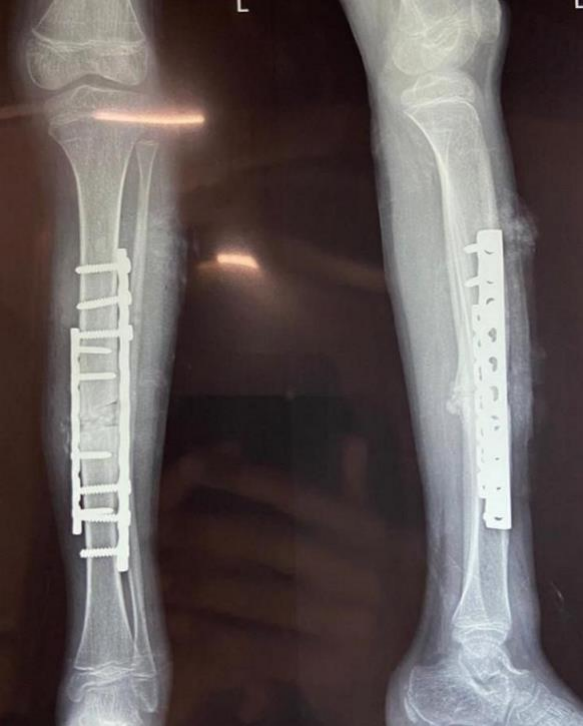
postoperative X-ray showing open reduction and internal fixation with plate osteosynthesis and bone grafting for midshaft tibia fracture

**Diagnostic assessment:** X-ray of the left leg was done, which shows midshaft tibia fracture with anterior bowing of tibia on the left side with osteogenesis imperfecta (Figure 3). Other investigations such as are complete blood count (CBC), liver function test (LFT), kidney function test (KFT), serum calcium, and serum phosphorus were also done.

**Diagnosis:** he was diagnosed with osteogenesis imperfecta with a midshaft tibia fracture on his left side.

**Therapeutic interventions:** the goal of treatment for osteogenesis imperfecta is to decrease pain and fracture risk while promoting mobility. To improve muscle strength, and improve mobility when recovering from fractures, physical therapy rehabilitation is very crucial for this patient. A physical rehabilitation program includes exercises to strengthen the upper limb and lower limb muscles. To help with day-to-day activities, physiotherapy may be required. The physical therapist follows a developmental sequence, beginning with head and trunk control, seated balance, and ambulation. To increase trunk control, strengthening of trunk and abdominal muscles were started. The easiest method to do this is to follow a graded progressive workout regimen that starts with prone and proceeds to sit. Rather than focusing solely on ambulation, treatment strategies should emphasize self-care and compensating strategies, which improve significantly at follow-up. The presence of intramedullary rods in the lower extremities indicates a poor prognosis for walking. As an outcome, rodding is a sign of a serious disorder. It is the most severe cases, intramedullary rodding in the lower limbs is used to support bone and repair abnormalities. To achieve ambulation, bracing, surgery and physical therapy are required also to strengthen muscles and increase the quality of life. The rehabilitation procedure must involve exercises such as range of motion (passively at first, then progressed to active assisted, active range of motion exercise), strengthening of lower limb muscles, balance and proprioception enhancement, gait and functional re-education. Once the cast is removed, at the post-operative health centre, or during physical therapy review, any progress in rehabilitation is re-evaluated under patient/family goals and surgeons' procedure. Walking in the community was bought to be the primary focus of rehabilitation for our patient.

**Figure 4:**
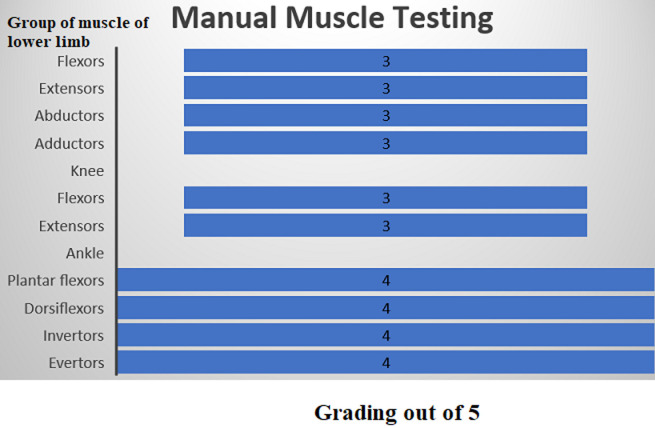
a funnel chart representing manual muscle testing (MMT) of the left lower limb according to medical research council (MRC) grading

**Follow-up and outcome of interventions:** follow-up was taken after the 4 weeks of physiotherapy rehabilitation which includes the range of motion, manual muscle testing, numerical pain rating scale, mini-mental state examination, lower extremity functional scale and paediatric quality of life inventory (Table 1, Table 2), (Figure 4).

**Table 1:** range of motion of both unaffected and affected lower limb

Joint	Right	Left
**Hip**		
Flexion	110	40
Extension	25	10
Abduction	40	35
Adduction	20	15
Medial Rotation	30	30
Lateral Rotation	40	55
**Knee**		
Flexion	100-0	85-0
Extension	0-100	0-85
**Ankle**		
Plantarflexion	20	15
Dorsiflexion	30	25

**Table 2:** pre and post rehabilitation findings of outcome measure, that are numeric pain rating scale, mini-mental state examination, lower extremity function test, pediatric quality of life inventory

Outcome measures	Pre	Post
Numeric pain rating scale (NPRS)	8/10	6/10
MMSE	17/30	22/30
LEFS	20/80	45/80
Peds QL (Parent report for children is used, as the patient is unable to speak)	64/92	70/92

**Informed consent:** for writing the case report, proper oral consent was obtained from the patient's father as the patient is unable to speak.

## Discussion

Osteogenesis imperfecta is an uncommon genetic disease characterized by skeletal anomalies that produce increased bone fragility, lower bone mass, deformity, and other connective-tissue signs in which the body is unable to form healthy bones. Osteogenesis imperfecta is a complicated condition defined by a variety of clinical manifestations as well as a genetic mutation. Treatment focuses on preventing or treating symptoms, which vary depending on the individual. Coxa vara, related defects, kyphoscoliosis, too much upper limb angulation trying to prevent the use of an auxiliary device, limb disparity, leg axis deviation, the earliest appearance of fractures, and the degree of bone fragility can all cause gait disorder. It was caused by gene mutations that disrupt collagen synthesis. The current method is based on a multidisciplinary method that takes rehabilitation, orthopedic surgery, and the use of bisphosphonates as a preventative measure. A study done by Hoyer-Kuhn H [8], on the patient's motor function and walking distance improve as a consequence of a particular rehabilitation method that increases mobility in children with osteogenesis imperfecta. Another study was done by Moreira CLM [9], on physiotherapy and patients with osteogenesis imperfecta that highlights successful physiotherapy and early encouragement to perform active movements within a safe environment, or even after fractures. Thus, many studies show that the patients who are suffering from OI need physical therapy rehabilitation which were beneficial. Physical therapy involves strengthening exercises, stretching exercises, bracing, functional activities, gait training, etc.

## Conclusion

This case study highlighted that physical therapy rehabilitation along with multidisciplinary care; can help the patient with pain management and functional independence which enhances the patient´s strength, and endurance, prevent deformity, and improves the patient quality of life.
